# Synthesis, Phase Behavior and Computational Simulations of a Pyridyl-Based Liquid Crystal System

**DOI:** 10.3390/molecules26216416

**Published:** 2021-10-24

**Authors:** Fowzia S. Alamro, Hoda A. Ahmed, Saheed A. Popoola, Asmaa Aboelnaga

**Affiliations:** 1 Department of Chemistry, College of Science, Princess Nourah Bint Abdulrahman University, Riyadh 11671, Saudi Arabia; fsalamro@pnu.edu.sa; 2 Department of Chemistry, Faculty of Science, Cairo University, Cairo 12613, Egypt; 3 Chemistry Department, College of Sciences, Taibah University, Yanbu 30799, Saudi Arabia; 4 Chemistry Department, Faculty of Science, Islamic University of Madinah, Al-Madinah Al-Munawwarah 42351, Saudi Arabia; abiodun@iu.edu.sa; 5 Department of Chemistry, Faculty of Women for Arts, Science and Education, Ain Shams University, Cairo 11566, Egypt; asmaa_aboelnaga77@yahoo.com

**Keywords:** pyridyl based liquid crystals, schiff base/ester, mesophase behavior, computational DFT calculations

## Abstract

A homologous set of liquid crystalline materials (**Tn**) bearing Schiff base/ester linkages were prepared and investigated via experimental and theoretical techniques. Terminal flexible groups of different chain lengths were connected to the end of phenylbenzoate unit while the other end of molecules was attached to the heterocyclic pyridine moiety. The molecular structures of the designed molecules were evaluated by FT-IR, NMR spectroscopic analyses, whereas their mesomorphic properties were investigated by polarized optical microscopy (POM) and differential scanning calorimetry (DSC). They all exhibited dimorphic properties with the exception of the members having the shortest and longest terminal flexible chains (*n* = 6 and 16), which were monomorphic. The **T16** derivative was further found possessing purely smectic A (SmA) mesophase while others have their lengths covered by nematic (N) phase. Moreover, the computational evaluation of the azomethine derivatives was carried out using a DFT approach. The polarity of the investigated derivatives was predicted to be appreciably sensitive to the size of the system. Furthermore, the Frontier molecular orbitals analysis revealed various distributions of electron clouds at HOMO and LUMO levels.

## 1. Introduction

Today, structure–activity relationship tools have been required to prepare the material in order to achieve proper characteristics for device applications [1,2,3,4]. Calamitic LCs are a prominent kind of mesogens that exhibit smectic and N phases due to their anisotropic self-association. As a result, the LC molecular architectures are designed using anisotropic mesogenic shape factors and principles. Several two- or three-ring compounds based on Schiff base/ester LCs have been reported and their optical behaviors were analyzed to obtain the correlation between the geometry of mesogens and their mesophase behaviors [5,6,7,8,9].

Due to their beneficial capacity to impart lateral and/or longitudinal dipoles, together with variations in their molecular geometries, which are reflected in their optical and electrical behaviors, heterocyclic-based LC materials have been of great interest to many researchers [10,11,12,13,14,15,16,17,18,19,20,21,22,23,24]. Pyridine exhibits a dipole moment of 2.19 D, which is about five-hundredths of that of a cyano group (~4.0 D) [25]; consequently, it influences the stability of the mesophase when it is utilized as a moiety within the mesogens [25,26,27,28,29,30]. Additionally, the pyridyl moiety is capable of forming intermolecular hydrogen bonding with components that have carboxylic units [31]. The insertion of new mesogenes or heterocyclic rings and changeable terminal groups will affect the atomic geometry and offer wide mesophase ranges of the resultant materials [32]. Moreover, slight modification within the molecular shape enables new significant changes within the phase transitions and plays an essential role in the kind and stability of the produced phase [33,34,35,36,37].

In order to investigate the structural linearity of synthesized molecules and molecular polarizability, as well as the geometrical parameters of each prepared compound, a DFT approach was used and the results were correlated with the experimental data [33,34,35,36,37,38,39,40]. It is essential to note that the DFT calculations assume a gas phase for all molecules, as such the most favorable predicted geometry could differ from that of empirical which occurs in a condensed phase such as LC mesophase, where more prolonged molecules are usually favored [41].

In this work, we synthesized and investigated the mesomorphic transitional properties of a three-ring homologous series based on Schiff base/ester mesogenic cores. The studied molecules have a heterocyclic pyridine ring in one terminal while the other end is connected to different flexible alkoxy chains. The influence of various alkoxy chains towards the mesomorphic properties of the derivatives was evaluated. Furthermore, important theoretical data were computed via the DFT method for the synthesized molecules and correlated with the experimental results.

## 2. Experimental

### 2.1. Synthesis of Materials

**Scheme 1:**
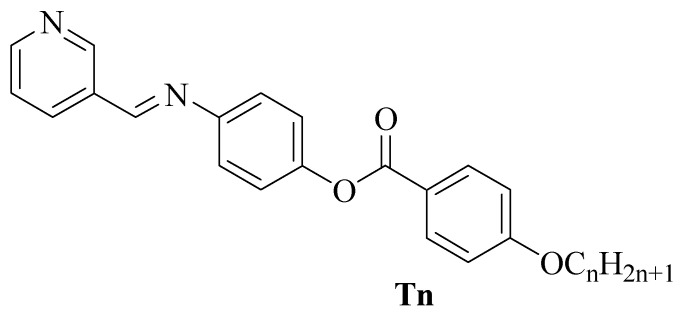

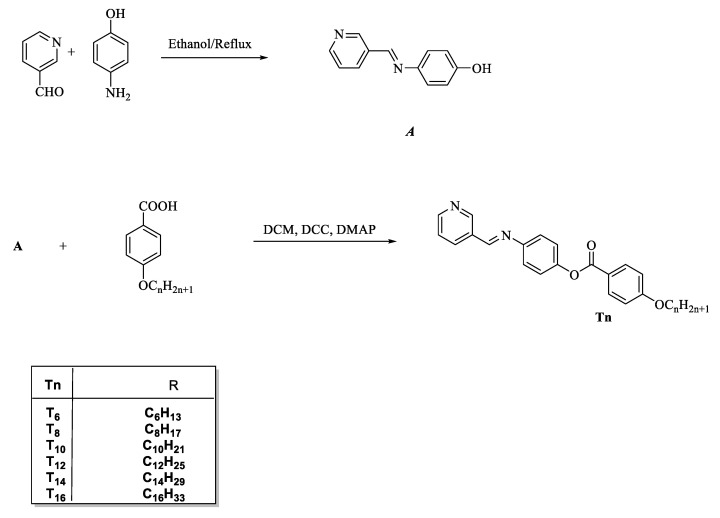
Synthesis of (*E*)-4-((pyridin-3-ylmethylene)amino)phenyl 4-(alkoxy) benzoate **Tn**. *n* = 6, 8, 10, 12, 14 and 16 carbons.

Compounds **T6**, **T8**, **T10**, **T12**, **T14** and **T16** were prepared according to Scheme 1:

Details of preparation and spectroscopic characterizations are given in Supplementary Information (Figures S1–S5).

(*E*)-4-((Pyridin-3-ylmethylene)amino)phenyl 4-(hexyloxy)benzoate (**T6**), yield: 90.7%; mp 111.0 °C, FTIR (ύ, cm^−1^): 2920, 2861 (CH_2_ stretching), 1719 (C=O), 1602 (C=N), 1463 (C-O_Asym_), 1259 (C-O_Sym_). ^1^H-NMR (300 MHz, CDCl_3_): *δ* = 0.89 (t, 3H, CH_3_(CH_2_)_3_CH_2_CH_2_, *J* = 7.5 Hz), 1.29–1.46 (m, 6H, CH_3_(CH_2_)_3_CH_2_CH_2_), 1.75 (q, 2H, *J* = 4.8 Hz, CH_3_(CH_2_)_3_CH_2_CH_2_), 4.07 (t, 2H, CH_3_(CH_2_)_3_CH_2_CH_2_, *J* = 6.75 Hz), 7.10 (d, 2H, *J* = 6.6 Hz, Ar-H), 7.32 (d, 2H, *J* = 6.3 Hz, Ar-H), 7.39 (d, 2H, *J* = 5.3 Hz, Ar-H), 7.56–7.84 (dd, 1H, py-H), 8.07 (d, 2H, *J* = 7.5 Hz, Ar-H), 8.32 (d, 1H, *J* = 6 Hz, py-H), 8.72 (d, 1H, *J* = 2.1 Hz, py-H), 8.77 (s, 1H, py-H), 9.07 (s, 1H, CH=N). ^13^C-NMR (125 MHz, DMSO-*d*_6_, CDCl_3_): *δ* = 164.74, 163.71, 159.25, 152.48, 150.94, 149.64, 149.05, 135.46, 132.50 (2C), 131.99 (2C), 124.55, 123.21 (2C), 122.59 (2C), 121.22, 115.15, 68.48, 31.44, 28.93, 25.57, 22.53, 14.37. MS (*m*/*z*): 402.49, Anal.Calcd for: C_25_H_26_N_2_O_3_: Found (Calc.): C, 74.58 (74.60); H, 6.50 (6.51); N, 6.94 (6.96).

(*E*)-4-((Pyridin-3-ylmethylene)amino)phenyl 4-(octyloxy)benzoate (**T8**), yield: 89.0%; mp 78.0 °C, FTIR (ύ, cm^−1^): 2916, 2869 (CH_2_ stretching), 1710 (C=O), 1594 (C=N), 1462 (C-O_Asym_), 1259 (C-O_Sym_). ^1^H-NMR (300 MHz, CDCl_3_): *δ* = 0.88 (t, 3H, CH_3_(CH_2_)_4_CH_2_CH_2_CH_2_, *J* = 5.5 Hz), 1.26–1.30 (m, 12H, CH_3_(CH_2_)_4_CH_2_CH_2_CH_2_), 1.43 (q, 2H, *J* = 7.25 Hz, CH_3_(CH_2_)_4_CH_2_CH_2_CH_2_), 1.78 (q, 2H, *J* = 7.25 Hz, CH_3_(CH_2_)_4_CH_2_CH_2_CH_2_), 4.06 (t, 2H, CH_3_(CH_2_)_4_CH_2_CH_2_CH_2_, *J* = 6.4 Hz), 7.13 (d, 2H, *J* = 6.8 Hz, Ar-H), 7.35 (d, 2H, *J* = 6.6 Hz, Ar-H), 7.41 (d, 2H, *J* = 5.5 Hz, Ar-H), 7.50–7.54 (dd, 1H, py-H), 8.17 (d, 2H, *J* = 7.7 Hz, Ar-H), 8.42 (d, 1H, *J* = 6.2 Hz, py-H), 8.75 (d, 1H, *J* = 2.8 Hz, py-H), 8.78 (s, 1H, py-H), 9.09 (s, 1H, CH=N). MS (*m*/*z*): 430.55, Anal.Calcd for: C_27_H_30_N_2_O_3_: Found (Calc.): C, 73.30 (75.32); H, 7.04 (7.02); N, 6.50 (6.51).

(*E*)-4-((Pyridin-3-ylmethylene)amino)phenyl 4-(decyloxy)benzoate (**T10**), yield: 89.0%; mp 79.0 °C, FTIR (ύ, cm^−1^): 2918, 2871 (CH_2_ stretching), 1712 (C=O), 1590 (C=N), 1462 (C-O_Asym_), 1255 (C-O_Sym_). ^1^H-NMR (300 MHz, CDCl_3_): *δ* = 0.85 (t, 3H, CH_3_(CH_2_)_6_CH_2_CH_2_CH_2_, *J* = 5.5 Hz), 1.24–1.40 (m, 12H, CH_3_(CH_2_)_6_CH_2_CH_2_CH_2_), 1.43 (q, 2H, *J* = 7.3 Hz, CH_3_(CH_2_)_6_CH_2_CH_2_CH_2_), 1.78 (q, 2H, *J* = 7.3 Hz, CH_3_(CH_2_)_6_CH_2_CH_2_CH_2_), 4.10 (t, 2H, CH_3_(CH_2_)_6_CH_2_CH_2_CH_2_, *J* = 6.0 Hz), 7.11 (d, 2H, *J* = 5.4 Hz, Ar-H), 7.33 (d, 2H, *J* = 6.3 Hz, Ar-H), 7.40 (d, 2H, *J* = 5.4 Hz, Ar-H), 7.57 (d, 1H, *J* = 7.5 Hz, py-H), 8.00 (d, 2H, *J* = 6.1 Hz, Ar-H), 8.32 (d, 1H, *J* = 7.5 Hz, py-H), 8.72 (d, 1H, *J* = 4.1 Hz, py-H), 8.77 (s, 1H, py-H), 9.09 (s, 1H, CH=N). MS (*m*/*z*): 562.71, Anal.Calcd for: C_29_H_34_N_2_O_3_: Found (Calc.): C, 75.97 (75.95); H, 7.45 (7.47); N, 6.09 (6.11).

(*E*)-4-((Pyridin-3-ylmethylene)amino)phenyl 4-(dodecyloxy)benzoate (**T12**), yield: 91.3%; mp 80.0 °C, FTIR (ύ, cm^−1^): 2922, 2873 (CH_2_ stretching), 1719 (C=O), 1586 (C=N), 1462 (C-OAsym), 1260 (C-OSym). 1H-NMR (300 MHz, CDCl3): δ = 0.86 (t, 3H, CH_3_(CH_2_)__8__CH_2_CH_2_CH_2_, *J* = 5.8 Hz), 1.25–1.254 (m, 16H, CH_3_(CH_2_)_8_CH_2_CH_2_CH_2_), 1.43 (q, 2H, *J* = 7.1 Hz, CH_3_(CH_2_)_8_CH_2_CH_2_CH_2_), 1.76 (q, 2H, *J* = 8.1 Hz, CH_3_(CH_2_)_8_CH_2_CH_2_CH_2_), 4.10 (t, 2H, CH_3_(CH_2_)_8_CH_2_CH_2_CH_2_, *J* = 6.4 Hz), 7.11 (d, 2H, *J* = 5.6 Hz, Ar-H), 7.33 (d, 2H, *J* = 6.6 Hz, Ar-H), 7.43 (d, 2H, *J* = 5.5 Hz, Ar-H), 7.59 (d, 1H, *J* = 7.7 Hz, py-H), 8.01 (d, 2H, *J* = 6.3 Hz, Ar-H), 8.32 (d, 1H, *J* = 7.6 Hz, py-H), 8.71 (d, 1H, *J* = 4.8 Hz, py-H), 8.77 (s, 1H, py-H), 9.06 (s, 1H, CH=N). MS (*m*/*z*): 590.33, Anal.Calcd for: C_3_1H_3_8N_2_O_3_: Found (Calc.): C, 76.50 (76.51); H, 7.77 (7.87); N, 5.78 (5.76).

(*E*)-4-((Pyridin-3-ylmethylene)amino)phenyl 4-(tetradecyloxy)benzoate (**T14**), yield: 92.0%; mp 88.0 °C, FTIR (ύ, cm^−1^): 2922, 2881 (CH_2_ stretching), 1717 (C=O), 1595 (C=N), 1466 (C-O_Asym_), 1259 (C-O_Sym_). ^1^H-NMR (300 MHz, CDCl_3_): *δ* = 0.86 (t, 3H, CH_3_(CH_2_)_10_CH_2_CH_2_CH_2_, *J* = 5.6 Hz), 1.25 (m, 20 H, CH_3_(CH_2_)_10_CH_2_CH_2_CH_2_), 1.43 (q, 2H, *J* = 7.7 Hz, CH_3_(CH_2_)_10_CH_2_CH_2_CH_2_), 1.74 (q, 2H, *J* = 6.8 Hz, CH_3_(CH_2_)_10_CH_2_CH_2_CH_2_), 4.11 (t, 2H, CH_3_(CH_2_)_10_CH_2_CH_2_CH_2_, *J* = 6.3 Hz), 7.11 (d, 2H, *J* = 5.1 Hz, Ar-H), 7.35 (d, 2H, *J* = 6.0 Hz, Ar-H), 7.39 (d, 2H, *J* = 5.4 Hz, Ar-H), 7.58 (d, 1H, *J* = 7.2 Hz, py-H), 8.06 (d, 2H, *J* = 6.6 Hz, Ar-H), 8.32 (d, 1H, *J* = 7.7 Hz, py-H), 8.71 (d, 1H, *J* = 4.4 Hz, py-H), 8.78 (s, 1H, py-H), 9.08 (s, 1H, CH=N). MS (*m*/*z*): 616036, Anal.Calcd for: C_33_H_42_N_2_O_3_: Found (Calc.): C, 77.00 (77.01); H, 8.24 (8.23); N, 5.45 (5.44).

(*E*)-4-((Pyridin-3-ylmethylene)amino)phenyl 4-(hexadecyloxy)benzoate (**T16**), yield: 94.0%; mp 93.0 °C, FTIR (ύ, cm^−1^): 2926, 2879 (CH_2_ stretching), 1726 (C=O), 1595 (C=N), 1452 (C-O_Asym_), 1253 (C-O_Sym_). ^1^H-NMR (300 MHz, CDCl_3_): *δ* = 0.85 (t, 3H, CH_3_(CH_2_)_12_CH_2_CH_2_CH_2_, *J* = 5.5 Hz), 1.24–1.40 (m, 24H, CH_3_(CH_2_)_6_CH_2_CH_2_CH_2_), 1.67 (q, 2H, *J* = 7.5 Hz, CH_3_(CH_2_)_12_CH_2_CH_2_CH_2_), 1.75 (q, 2H, *J* = 7.5 Hz, CH_3_(CH_2_)_12_CH_2_CH_2_CH_2_), 4.07 (t, 2H, CH_3_(CH_2_)_12_CH_2_CH_2_CH_2_, *J* = 6.2 Hz), 6.58 (d, 2H, *J* = 5.4 Hz, Ar-H), 6.88 (d, 2H, *J* = 6.3 Hz, Ar-H), 7.07 (d, 2H, *J* = 5.4 Hz, Ar-H), 7.41 (d, 1H, *J* = 7.5 Hz, py-H), 8.24 (d, 1H, *J* = 7.5 Hz, py-H), 8.26 (d, 8.86 (d, 1H, *J* = 4.1 Hz, py-H), 8.87 (s, 1H, py-H), 9.09 (s, 1H, CH=N). MS (*m*/*z*): 646.88, Anal.Calcd for: C_35_H_46_N_2_O_3_: Found (Calc.): C, 77.44 (77.45); H, 8.53 (8.54); N, 5.15 (5.16).

### 2.2. Computational Details

The geometry of the **Tn** compound series studied was fully optimized without geometrical restriction using the GAUSSIAN 09 program (Wallingford, CT 06492 USA) [42]. Frequency calculation was later carried out to establish the convergence nature of the compounds and all the predicted frequencies were found to be real. Furthermore, both Frontier molecular orbitals and the molecular electrostatic potential (MEP) surfaces were generated from the check files (.chk) of optimized molecules. All the calculations were executed using density functional theory (DFT) by employing the B3LYP method [43,44] while utilizing 6–31g(d,p) as the basis set.

## 3. Results and Discussion

### 3.1. Mesomorphic Behavior

**Figure 1:**
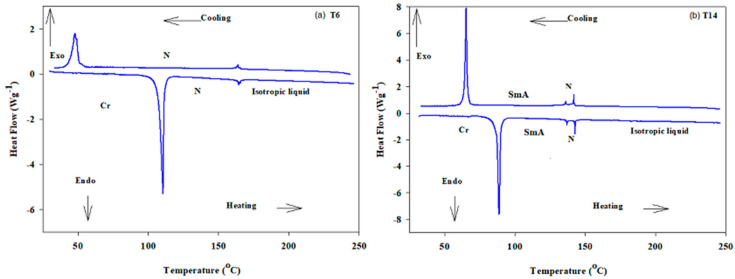
DSC thermograms of (**a**) **T6** and (**b**) **T14** samples.

**Figure 2:**
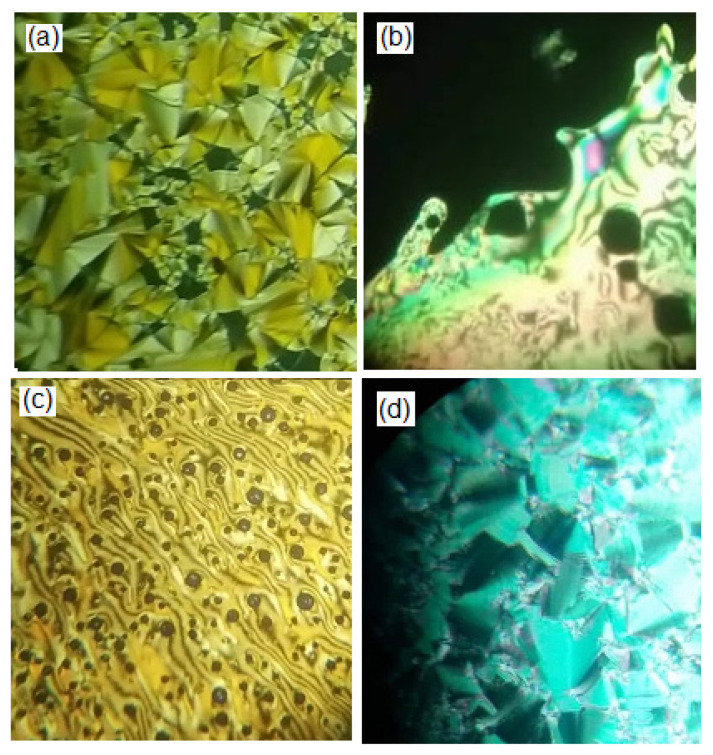
POM textures via heating of (**a**) SmA phase at 102 °C for **T14** derivative; (**b**) N phase at 150 °C for **T14** derivative; (**c**) N phase at 130 °C for **T6** derivative and (**d**) SmA phase at 110 °C for **T16** derivative.

**Figure 3:**
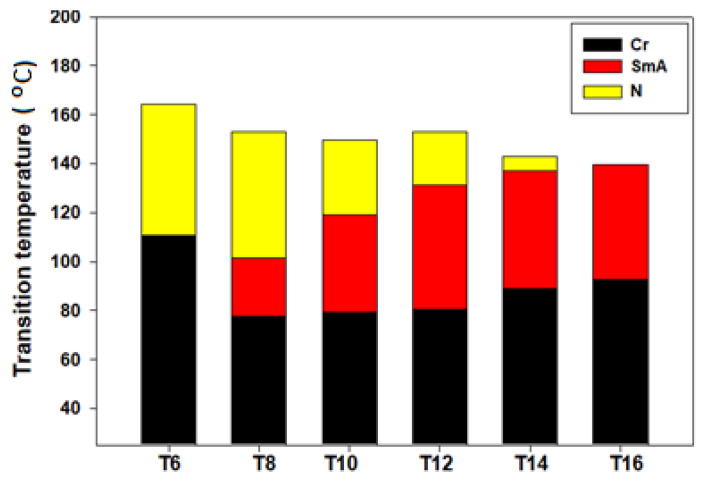
Effect of terminal length on the mesomorphic behavior of **Tn** series.

Representative examples of DSC thermograms for derivatives **T6** and **T14** via both heating and cooling rounds are illustrated in Figure 1. Designed compounds (Figure 1a,b) showed two or three endotherm peaks of transitions during the heating cycle and exothermic transitions during the cooling round, depending on the number and type of formed mesophases according to the corresponding length of the attached terminal alkoxy chain. The POM analysis revealed images which confirmed smectic A and N mesophases (Figure 2) depending on n. The N phase showed threads/a schlieren image and the SmA phase had a focal conic fan texture. The synthesized materials have enantiotropic behavior. Results of the mesomorphic transitions (temperatures, enthalpy and mesophase range) of all the investigated series (**Tn**), as measured from DSC analyses, are collected in Table 1. Moreover, the transition temperatures of all samples were graphically illustrated in Figure 3 in order to investigate the impact of terminal attached flexible chain length on the mesophase property of the series.

The results presented in Table 1 and Figure 3 revealed that the melting transitions have an irregular manner with the increasing number of carbons in the terminal chain (*n* = 6 to 16). Melting temperatures are related to the polarizability and molecular shape of the designed compounds [45]. Moreover, all the compounds of the group (**Tn**) are enantiotropic with a high good mesomorphic range and thermal stability. For the shortest chain derivative (**T6**), it has a monomorphic property exhibiting purely nematogenic phase. The N mesophase stability and range for **T6** are 164.4 and 53.9 °C, respectively. By lengthening the terminal alkoxy chain from *n* = 6 to 8, the SmA starts to appear and the N phase stability decreases to 153.1 °C together with the N range, which descends to 51.5 °C for **T8**. Compounds **T10**, **T12** and **T14** are also found to be dimorphic, exhibiting SmA and N mesophases. The SmA range and stability increases with the increasing terminal length of flexible chain *n* from 8 to 14 carbons, but the reverse is the case for the N phase thermal stability and range (Table 1). The resultant phase becomes purely smectogeic in the compound with the longest chain in the series (**T16**). Data suggest that **T16** derivative possesses only a monomorphic SmA mesophase with thermal stability and range of 139.5 and 46.6 °C, respectively. In general, the stability of the N phase decreases while the SmA phase increases with increasing terminal chain length [46,47]. The reduction trend in the N phase thermal transition may be due to the rigid mesogen dilution. Nevertheless, the production of SmA phase reduced the nematogenic range as the alkoxy chain length increases. This could be attributed to the increment of the van der Waals forces of attraction within the long terminal chains that facilitated the lamellar packing for the appearance of the smectic phase.

The normalized entropy changes (**∆*S***/R) of the produced mesophases were estimated for all samples and summarized in Table 2. This was found to be independent of the size of the system as random trend and little magnitudes of the **∆*S***/R related to the SmA-N, N-I transitions were observed. However, the observed little values in all materials can be due to some degree of biaxiality formed by the ester group, which in return decreases the SmA-N, N-I entropy changes [48,49,50]. The alteration in the entropy changes with alkoxy terminal chains may be ascribed to the interactions between molecules, which are influenced by dipole moment, polarizability, inflexibility, length/breadth proportion and the structural shape of the molecules. These structural parameters may contribute to the conformational, translational and orientational entropies of the molecule in different magnitudes. In spite of the fact that the increment of alkoxy chain length has dilution impact of core/core interactions, it raises the polarizability of the whole molecule, which in return increases the intermolecular strengths between adjoining molecules that advances the degree of molecular ordering. The increment of **∆*S***/R values with the expansion of the carbon numbers in a flexible chain is likely due to the diminishing of the long orientational arrangement and the increment of the conformational number dispersions at the mesophase transitions.

### 3.2. Computational DFT Calculations and Geometrical Parameters

#### 3.2.1. Reactivity Parameters

**Figure 4:**
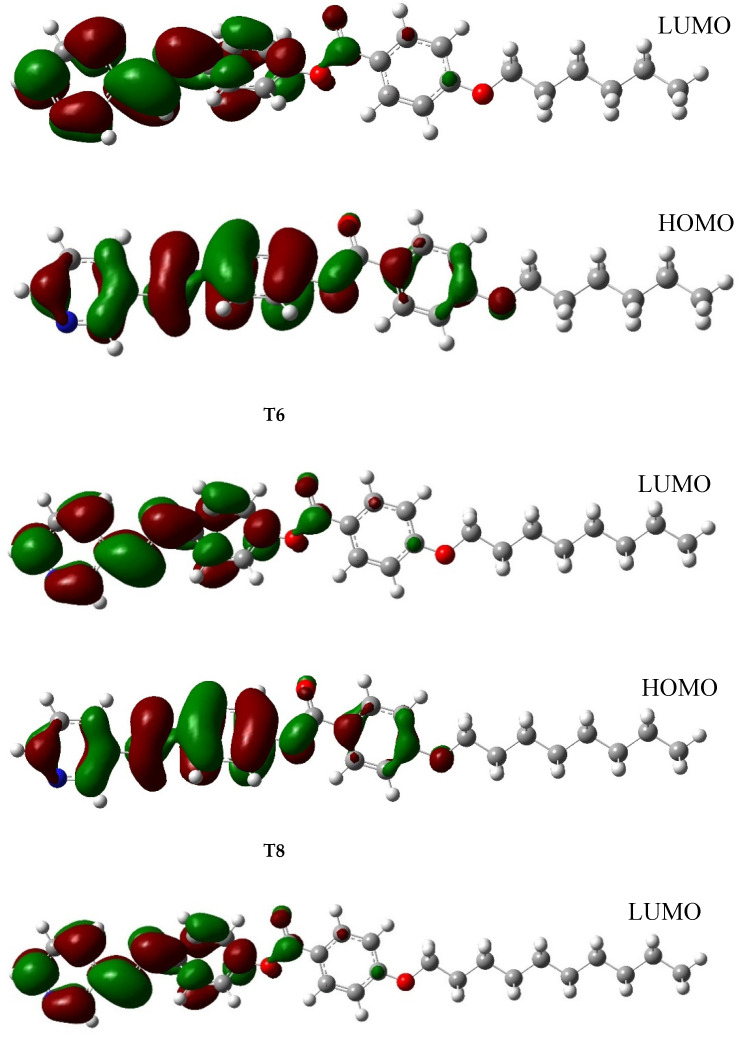

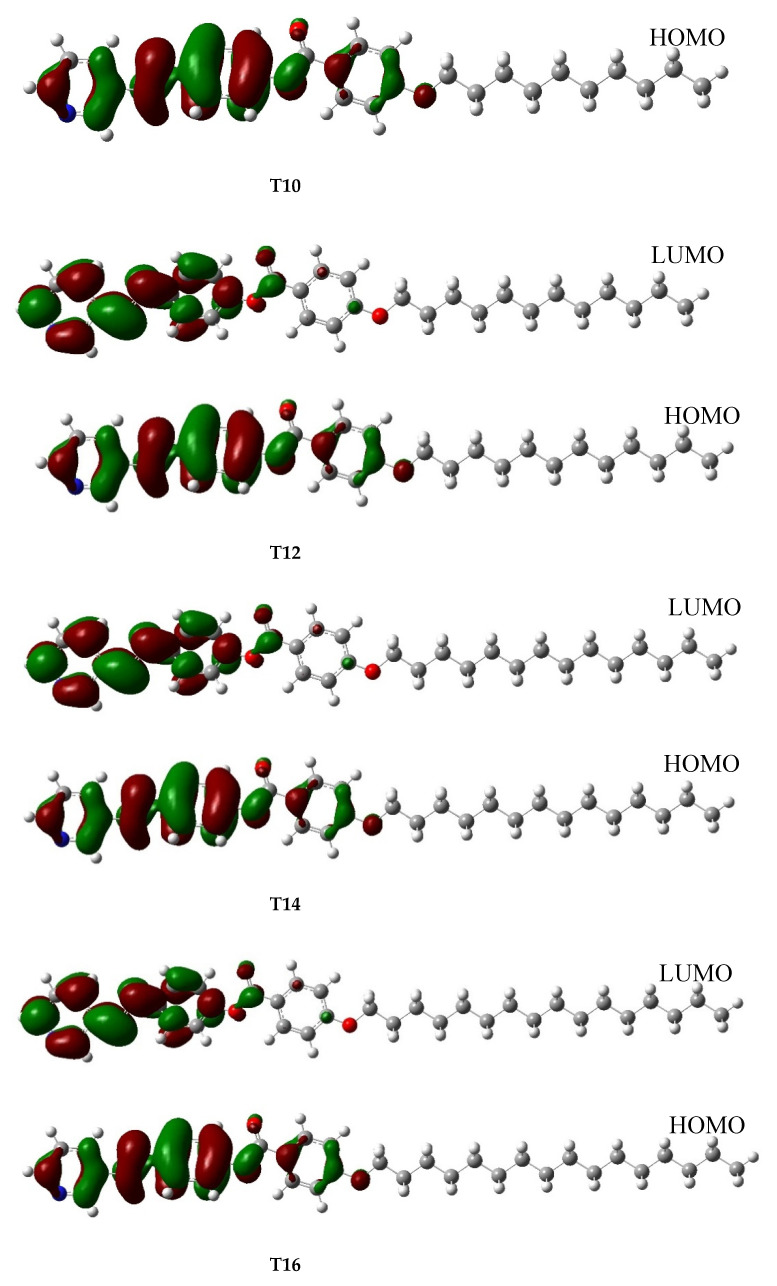
Frontier molecular orbitals for the compound **Tn** calculated at the B3LYP/6–31g(d,p) level.

**Figure 5:**
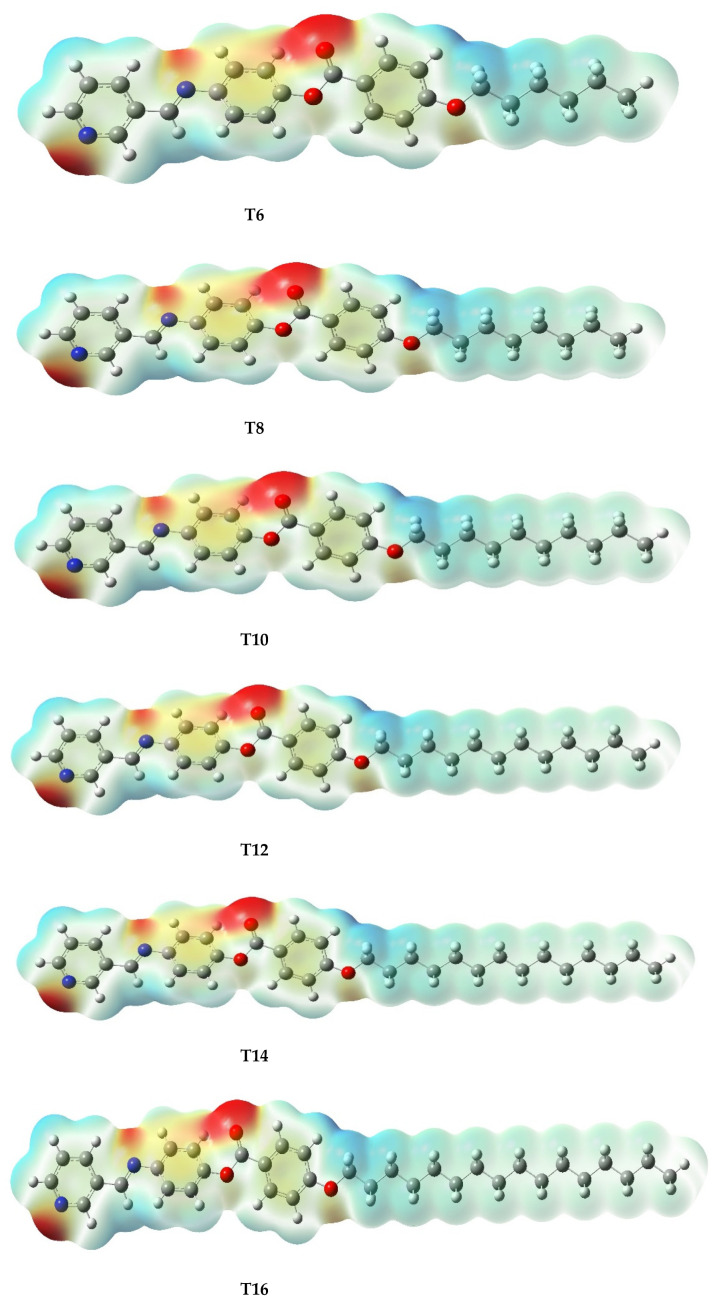
Molecular electrostatic potential (MEP) recorded at an isovalue of 0.001 and calculated at the B3LYP/6–31g(d,p) level for the **Tn** series.

The reactivity of chemical compounds is usually inferred from the HOMO–LUMO energy gap (∆E) together with the ionization potential (I.P) and electron affinity (EA) [51,52]. Following the optimization calculation, the reactivity parameters highlighted in Table 3 were computed for the Tn series. The result shows similar chemical reactivity for all the derivatives as the same values were predicted for the corresponding reactivity indicators. This suggests that the size of the system does not play a significant role in the reactivity of the series. However, the polarity of the derivatives seems to be a bit sensitive to the size of the system as the calculated dipole moment listed in Table 3 and the dipole moment vectors (x and z directions) portrayed in Figure S6 gradually increased with the increasing alkoxy chain length. Regarding the Frontier molecular orbital depicted in Figure 4, the similar HOMO and LUMO distributions recorded for the all the derivatives could be attributed to the nearly equal corresponding HOMO and LUMO energy levels highlighted in Table 3 [52]. On the part of HOMO, the electron clouds were predicted to be evenly allocated over the carbon atoms and the π-electrons system of the pyridyl ring and its immediate phenyl as well as their C=N linkage. Moreover, an appreciable cloud distribution was recorded over the second phenyl ring carbon and its π-electrons together with the alkoxy oxygen. On the other hand, the second phenyl ring and the alkoxy oxygen at the LUMO level were found to be electron deficient while electron clouds distribution only covered the carbon atoms of the pyridyl and the first phenyl rings as well as their C=N linkage. On the part of the molecular electrostatic potential (MEP) presented in the Figure 5, the carbonyl oxygen of the ester linkage in each of the **Tn** series has high electron density but low electrostatic potential as reflected by the red cloud over its region [53,54,55]. Similarly, an appreciable electron density was observed for the pyridyl nitrogen heteroatom, C=N linkage nitrogen atom and alkoxyl oxygen of the derivatives. It could be concluded that the kind and the thermal stability of the formed phases facilitate the molecular space-filling due to the attached terminals that influence the physical geometrical parameters to enhance the intermolecular attractions between molecules. Moreover, the high electron density predicted for the carbonyl oxygen of –OCO- linkage together with the appreciable quantity recorded for that of -C=N- linkage, as reflected in Figure 5, suggests significant contribution of these linkages to the derivatives’ polarizability, which is an important factor that increases the mesophase thermal stability of a compound [56,57].

#### 3.2.2. Energy

The calculated zero-point energy and thermal energy, as well as the thermodynamic parameters listed in Table 4, were calculated to increase with the size of the derivative in the series. This result quite agrees with reports in the literature as the energy is an extensive property [54,55]. According to McMillan relation [58,59] the enthalpy change associated with the Smectic to nematic transition (Table 1) increases as the width of the N phase reduces. Theoretically, the predicted enthalpy changes for molecules increase with the terminal chain length (n). Furthermore, the calculated thermal energy accents to the thermal stability highlighted for the SmA-N transition of the derivatives in Table 1 were recorded to increase with size of the system. In the same vein, the predicted entropy is consistent with the normalized entropy (Table 2) obtained for the **Tn** series.

## 4. Conclusions

New mesomorphic pyridyl series’ with -CH=N- and -OCO- connecting linkages, (*E)-4-((pyridin-3-ylmethylene)amino)phenyl 4-(alkoxy) benzoate*, were synthesized and experimentally as well as theoretically investigated. Molecular structures were elucidated using elemental analyses, FT-IR and ^1^H-NMR spectroscopy. Liquid crystalline activities of prepared derivatives were examined by DSC and POM analyses. Theoretical simulations were carried out by DFT calculation method.

The study revealed that:

- All designed heterocyclic compounds exhibit good thermal mesomorphic stability with enantiotropic transitions.
- The N mesophase covers all lengths of the series except the longest chain member (*n* = 16) exhibiting purely a smectogenic phase.
- Geometrical simulation parameters of the formed derivatives are highly affected by the mesomeric nature of the pyridyl moiety and the terminal extremes of alkoxy chains.
- The polarity and polarizability of the investigated derivatives seem to be a bit sensitive to the length of the designed system.
- The Frontier molecular orbital analysis shows even distribution of electron clouds over the carbon atoms and the π-electrons system of the pyridyl and phenyl rings together with C=N linkage at both HOMO and LUMO levels.
- The MEP affirm the carbonyl oxygen of the ester linkage to be of high electron density but low electrostatic potential.
- The calculated thermal energy accents to the experimental values of thermal stability were recorded to increase with size of the system.

## Figures and Tables

**Table 1:** Phase transition temperatures (°C) and (enthalpy of transitions in kJ/mol) of **Tn**.

Compounds	*T*_Cr-SmA_	*T*_Cr-N_	*T*_SmA-N_	*T*_SmA-I_	*T*_N-I_	∆*T*_SmA-N_	∆*T*_SmA_	∆*T*_N_
**T6**		110.5 (40.56)	-	-	164.4 (1.46)	-	-	53.9
**T8**	77.8 (34.43)	-	101.6 (1.55)	-	153.1 (1.23)	23.8	-	51.5
**T10**	79.3 (37.03)	-	118.9 (1.43)	-	149.7 (1.31)	39.6	-	30.8
**T12**	80.4 (55.4)	-	131.2 (1.91)	-	153.1 (1.44)	50.8	-	21.9
**T14**	88.9 (53.22)	-	137.2 (2.18)	-	142.9 (1.74)	48.3	-	5.7
**T16**	92.9 (57.51)	-	-	139.5 (2.07)	-	-	46.6	-

Cr-SmA = crystal to the SmA mesophase; Cr-N = crystal to the N mesophase; SmA-N = SmA to the N mesophase; SmA-I = SmA to the isotropic mesophase; N-I = nematic to the isotropic mesophase.

**Table 2:** Normalized entropy changes of transitions for present series **Tn**.

Compounds	∆*S*_SmA-N_/R	∆*S*_SmA-I_/R	∆*S*_N-I_/R
**T6**	-	-	0.40
**T8**	0.50	-	0.35
**T10**	0.44	-	0.37
**T12**	0.57	-	0.41
**T14**	0.64	-	0.49
**T16**	-	0.6	-

**Table 3:** Reactivity and polarity parameters of the **Tn** series calculated at the B3LYP/6–31g(d,p) level.

Compound	E_HOMO_ (eV)	E_LUMO_ (eV)	∆E (eV)	Dipole Moment (Debye)	I.P (eV)	E.A (eV)	Polarizability (Bohr^3^)
**T6**	−5.8926	−1.8294	4.0632	4.2156	5.8926	1.8294	333.3200
**T8**	−5.8902	−1.8286	4.0616	4.2622	5.8902	1.8286	356.7500
**T10**	−5.8894	−1.8283	4.0610	4.2873	5.8894	1.8283	379.8900
**T12**	−5.8891	−1.8281	4.0610	4.3020	5.8891	1.8281	402.9000
**T14**	−5.8888	−1.8281	4.0608	4.3110	5.8888	1.8281	425.8300
**T16**	−5.8885	−1.8278	4.0608	4.3170	5.8885	1.8278	448.7200

**Table 4:** Zero-point energy, thermal energy and thermodynamic parameters in kcalmol^−1^ for the compound **Tn** series calculated at the B3LYP/6–31g(d,p) level.

Compound	ZPE	Thermal	Enthalpy	Gibbs	Entropy
**T6**	289.4371	307.0444	307.6374	248.3823	198.7430
**T8**	325.2554	344.5532	345.1456	281.8461	212.3080
**T10**	361.0542	382.0526	382.6449	315.1104	226.5120
**T12**	396.8505	419.5507	420.1430	348.3339	240.8490
**T14**	432.6443	457.0482	457.6405	381.5380	255.2480
**T16**	468.4387	494.5450	495.1380	414.7377	269.6630

## Data Availability

The data presented in this study are available on request from the corresponding author.

## Supplementary Materials

The following are available online: Figure S1: ^1^H-NMR spectrum of **T6**; Figure S2: ^13^C-NMR spectrum of **T6**; Figure S3: ^1^H-NMR spectrum of **T10**; Figure S4: ^1^H-NMR spectrum of **T12**; Figure S5: ^1^H-NMR spectrum of **T14**; Figure S6: Atomic charges and dipole moment vectors, calculated at B3LYP/6–31G(d,p) level for the **Tn** series.

## Sample Availability

Samples of the compounds are available from the authors.
