# Supplementary material for: Synthesis, Phase Behavior and Computational Simulations of a Pyridyl-Based Liquid Crystal System
Source: Molecules. 2021 Oct 24;26(21):6416. doi: 10.3390/molecules26216416 (PMC8588046; doi:10.3390/molecules26216416)
Supplement: Supplementary file 1 [file molecules-26-06416-s001.zip › molecules-1419824-supplementary.pdf]

Supplementary Materials

Synthesis, phase behavior and computational simulations of pyridyl based liquid crystal system

Fowzia S. Alamro [1], Hoda A. Ahmed [2,3,*], Saheed A. Popoola [4], and Asmaa Aboelnaga [5]

[1] Department of Chemistry, College of Science, Princess Nourah bint Abdulrahman University, Riyadh 11671, Saudi Arabia; fsalamro@pnu.edu.sa
[2] Department of Chemistry, Faculty of Science, Cairo University, Cairo 12613, Egypt
[3] Chemistry Department, College of Sciences, Yanbu, Taibah University, Yanbu 30799, Saudi Arabia
[4] Chemistry Department, Faculty of Science, Islamic University of Madinah, Al-Madinah Al-Munawwarah 42351, Saudi Arabia; abiodun@iu.edu.sa
[5] Department of Chemistry, Faculty of Women for Arts, Science and Education, Egypt, Ain Shams University, Cairo, P.O. Box 11566, Egypt; asmaa_aboelnaga77@yahoo.com
* Correspondence: (H.A.A.) ahoda@sci.cu.edu.eg

1. *Materials*

Ethyl 4-aminobenzoate and nicotinaldehyde obtained from Sigma-Aldrich (Germany). 4-hydroxyaniline and 1-bromoalkanes, were obtained from Merck (Germany). *N,N'*-Dicyclohexylcarbodiimide (DCC) and 4–dimethylaminopyridine (DMAP) were purchased from Aldrich (Milwaukee, WI, USA). All chemicals were used without further purification. All the solvents used such as dichloromethane, ethanol, and methanol were of pure grade and purchased Aldrich.

*2. Preparation of (E)-4-((pyridin-3-ylmethylene)amino)phenol (***A***)*

(0.5 g, 4.1 mmol) of nicotinaldehyde mixed with equimolar equivalents of the and 4-hydroxyaniline (0.45 g, 4.1 mmol) in EtOH (10 ml) and refluxed for 2 h. The mixture was cooled to room temperature and filtered. The obtained solid was washed with cold EtOH and recrystallized twice from hot EtOH to give pure compounds, as indicated by thin-layer chromatography (TLC) analysis; mp = 204.0°C.

3. Preparation of **(***E)-4-((pyridin-3-ylmethylene)amino)phenyl 4-(alkoxy) benzoate* **Tn**

Molar equivalents of the *(E)-4-((pyridin-3-ylmethylene)amino)phenol (***A***)* and *4-(alkoxy)benzoic acid* (0.01 mol each) were dissolved in 25 ml dry methylene chloride (DCM). To the resulting mixture, DCC (0.02 mol) and few crystals of 4-(dimethylamino) pyridine (DMAP), as catalyst, were added and the solution left to stand for 72 h at room temperature with

continuous stirring. The solid was separated then filtered off and the solution evaporated. The solid residue obtained was recrystallized twice from acetic acid and twice from ethanol to give TLC pure products. The purity of the prepared samples was checked with TLC using TLC sheets coated with silica gel (E Merck), and CH_2Cl_2/CHOH (9:1) as eluent, whereby only one spot was detected by a UV-lamp.

4. *Characterization*

IR spectra of all investigated compounds **I***n*$_{a\text{-}d}$ were measured by Perkin-Elmer B25 (Perkin-Elmer, Inc., Shelton, CT USA) spectrophotometer. ^{1}H NMR spectra were performed using a Varian EM 350L 300 MHz spectrometer (Oxford, UK) using $CDCl_3$ as solvent the chemical shift values recorded as δ (ppm units). Elemental analyses for final products were carried out on Thermo Scientific Flash 2000 CHS/O Elemental Analyzer (Milan, Italy). Mass spectra were performed with GCMS-QP1000EX Mass spectrometer (Shimadtzu, Japan) and microanalyses with a Perkin-Elmer Series II 2400-CHN Analyser (Shelton, CT USA). The UV–vis absorption spectra were measured on a UV-1800 SHIMADZU (Japan) spectrophotometer in dichloromethane solutions.

Differential Scanning Calorimeter, TA instrument Co. Q20 (DSC; USA), was used for calorimetric measurements. The melting point and enthalpy of indium and lead was used for DSC calibration. Aluminum pans and 2–3 mg sample amounts were used for DSC measurements investigation. 30 ml/min nitrogen gas inert atmosphere and 10°C/min heating rate were selected for all measurements and all transitions were recorded from the heating scan. The texture of the mesophases was identified by a polarized light microscope (POM, Wild, Germany) with Mettler FP82HT hot stage. The transitions from liquid crystal phase to isotropic liquid under POM for all prepared derivatives were in all cases quite sharp, and there was no indication whatsoever that any of the transitions took place over a range of temperature.

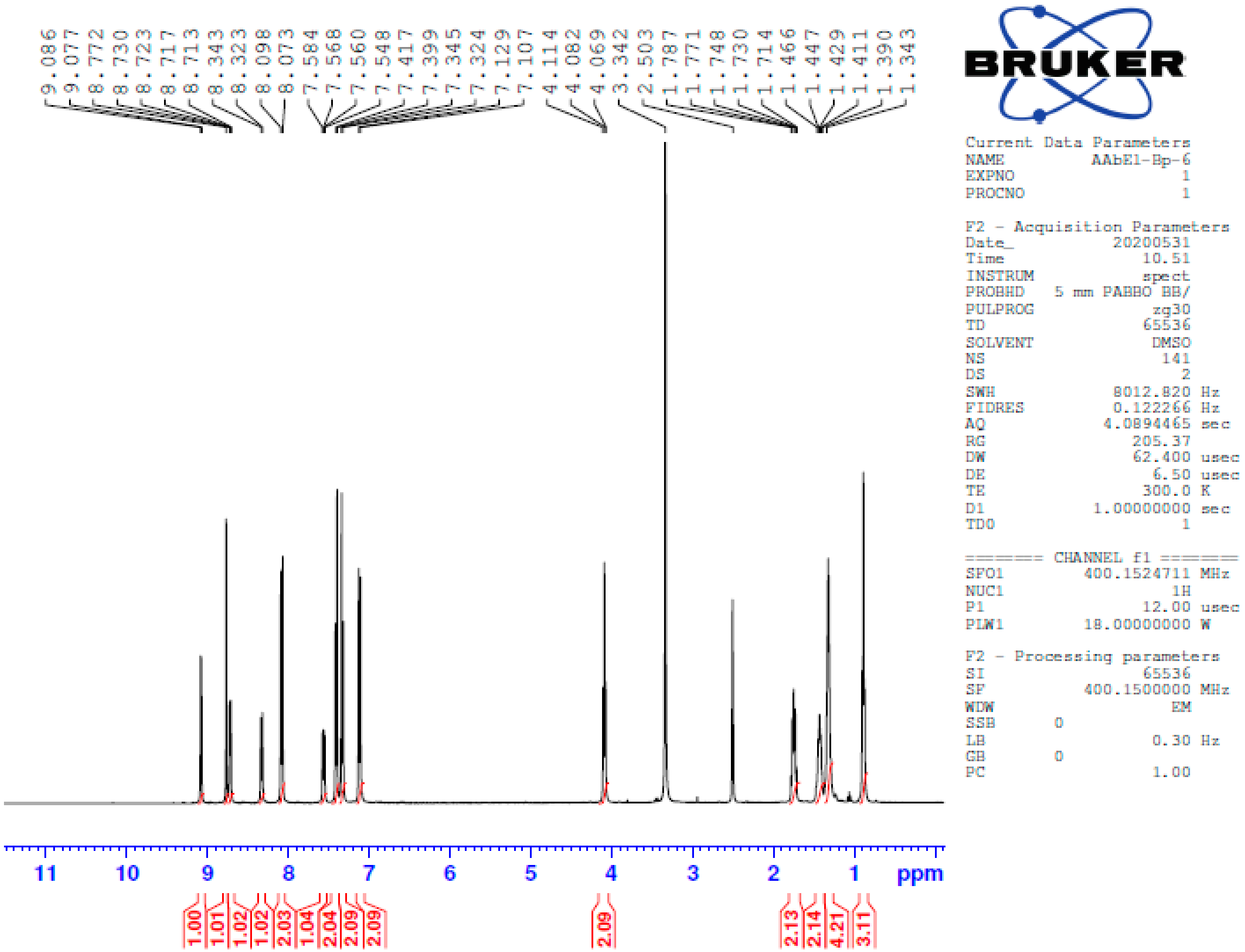

Figure S1.^{1}H-NMR spectrum of T6

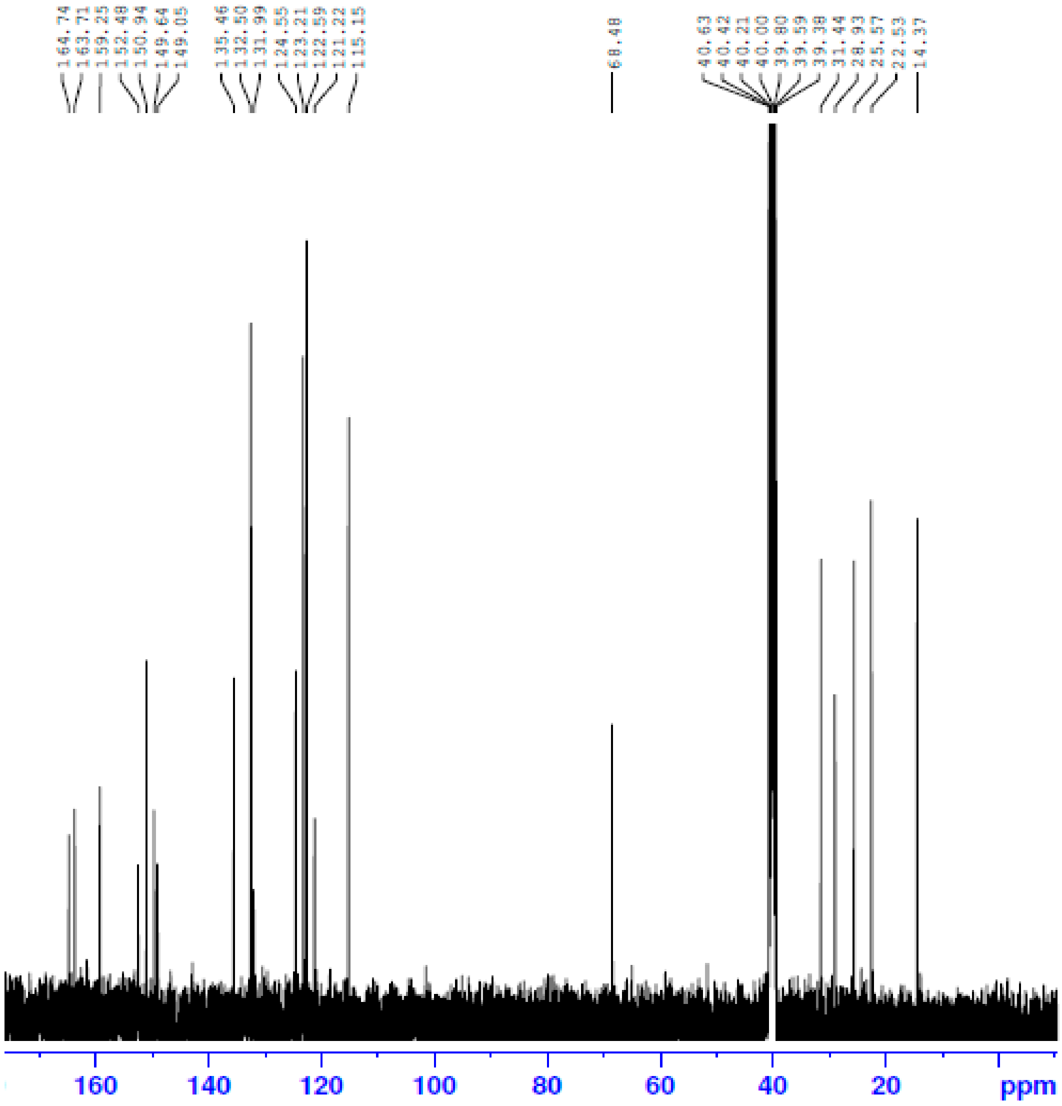

BRUKER

Current Data Parameters
NAME AAbE1-Bp-6
EXPNO 2
PROCNO 1

F2 - Acquisition Parameters
Date_ 20200531
Time 12.44
INSTRUM spect
PROBHD 5 mm PABBO BB/
PULPROG zgpg30
TD 65536
SOLVENT DMSO
NS 1471
DS 4
SWH 24038.461 Hz
FIDRES 0.366798 Hz
AQ 1.3631488 sec
RG 205.37
DW 20.800 usec
DE 6.50 usec
TE 300.0 K
D1 2.00000000 sec
D11 0.03000000 sec
TD0 1

======= CHANNEL f1 =======
SFO1 100.6278588 MHz
NUC1 13C
P1 10.00 usec
PLW1 47.00000000 W

======= CHANNEL f2 =======
SFO2 400.1516006 MHz
NUC2 1H
CPDPRG[2 waltz16
PCPD2 90.00 usec
PLW2 18.00000000 W
PLW12 0.34722000 W
PLW13 0.28125000 W

F2 - Processing parameters
SI 32768
SF 100.6177975 MHz
WDW EM
SSB 0
LB 1.00 Hz
GB 0
PC 1.40

Figure S2.^{13}C-NMR spectrum of T6

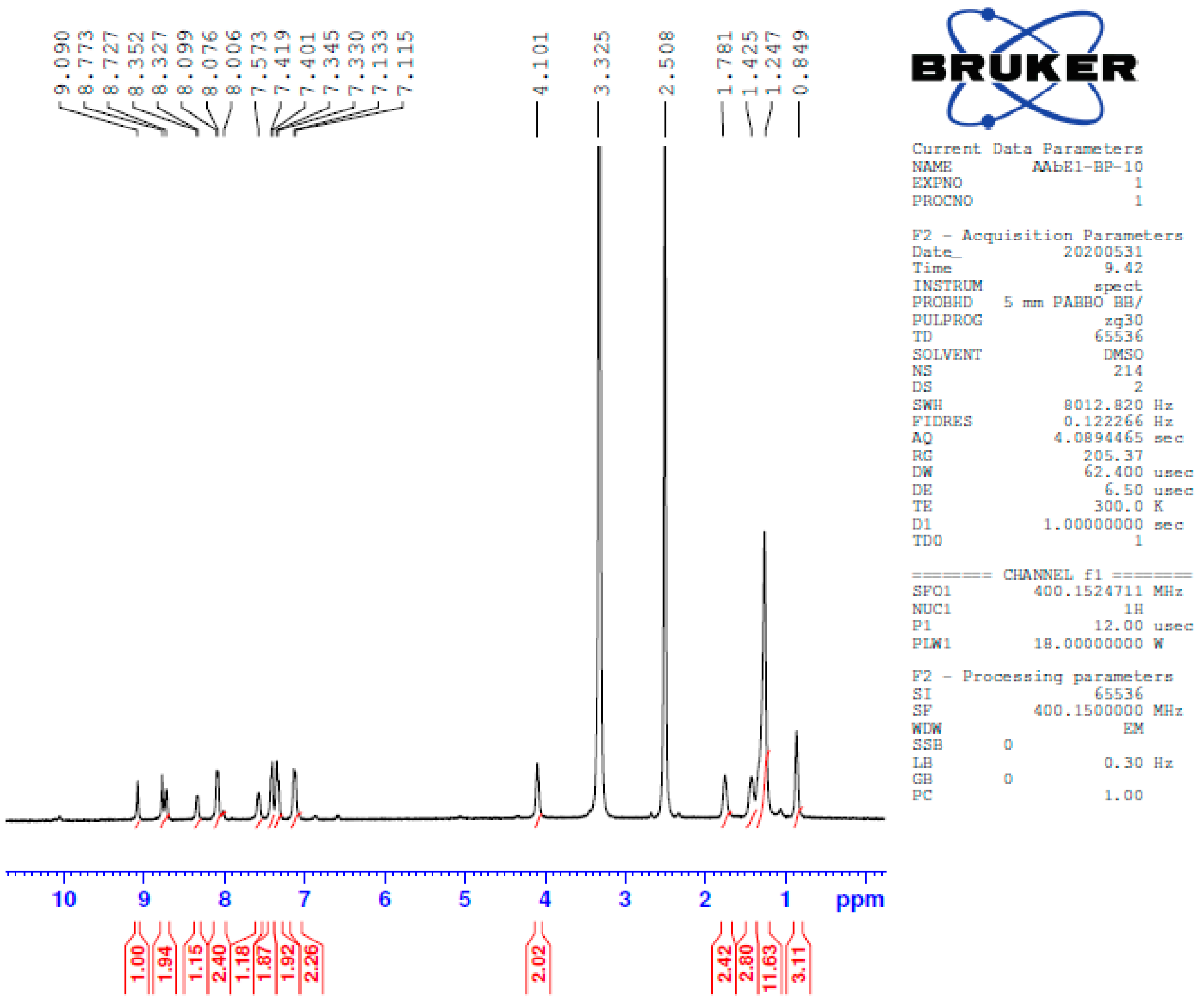

Figure S3.^{1}H-NMR spectrum of T10

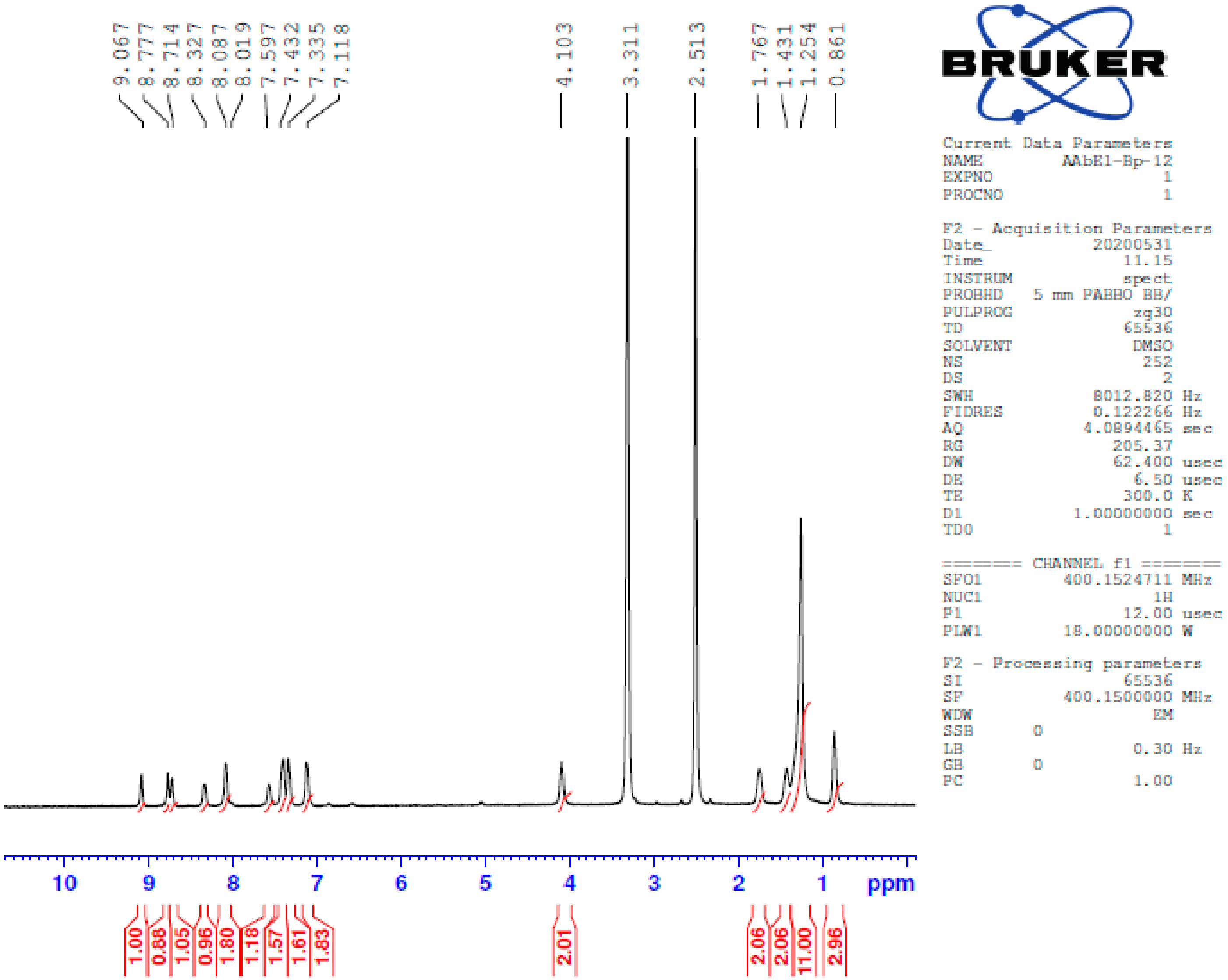
9.067
8.777
8.714
8.327
8.087
8.019
7.597
7.432
7.335
7.118
4.103
3.311
2.513
1.767
1.431
1.254
0.861
10
9
8
7
6
5
4
3
2
1
ppm
1.00
0.88
1.05
0.96
1.80
1.18
1.57
1.61
1.83
2.01
2.06
2.06
11.00
2.96
BRUKER
Current Data Parameters
NAME AAbE1-Bp-12
EXPNO 1
PROCNO 1
F2 - Acquisition Parameters
Date_ 20200531
Time 11.15
INSTRUM spect
PROBHD 5 mm PABBO BB/
PULPROG zg30
TD 65536
SOLVENT DMSO
NS 252
DS 2
SWH 8012.820 Hz
FIDRES 0.122266 Hz
AQ 4.0894465 sec
RG 205.37
DW 62.400 usec
DE 6.50 usec
TE 300.0 K
D1 1.00000000 sec
TD0 1
======= CHANNEL f1 =======
SFO1 400.1524711 MHz
NUC1 1H
P1 12.00 usec
PLW1 18.00000000 W
F2 - Processing parameters
SI 65536
SF 400.1500000 MHz
WDW EM
SSB 0
LB 0.30 Hz
GB 0
PC 1.00

Figure S4.^{1}H-NMR spectrum of T12

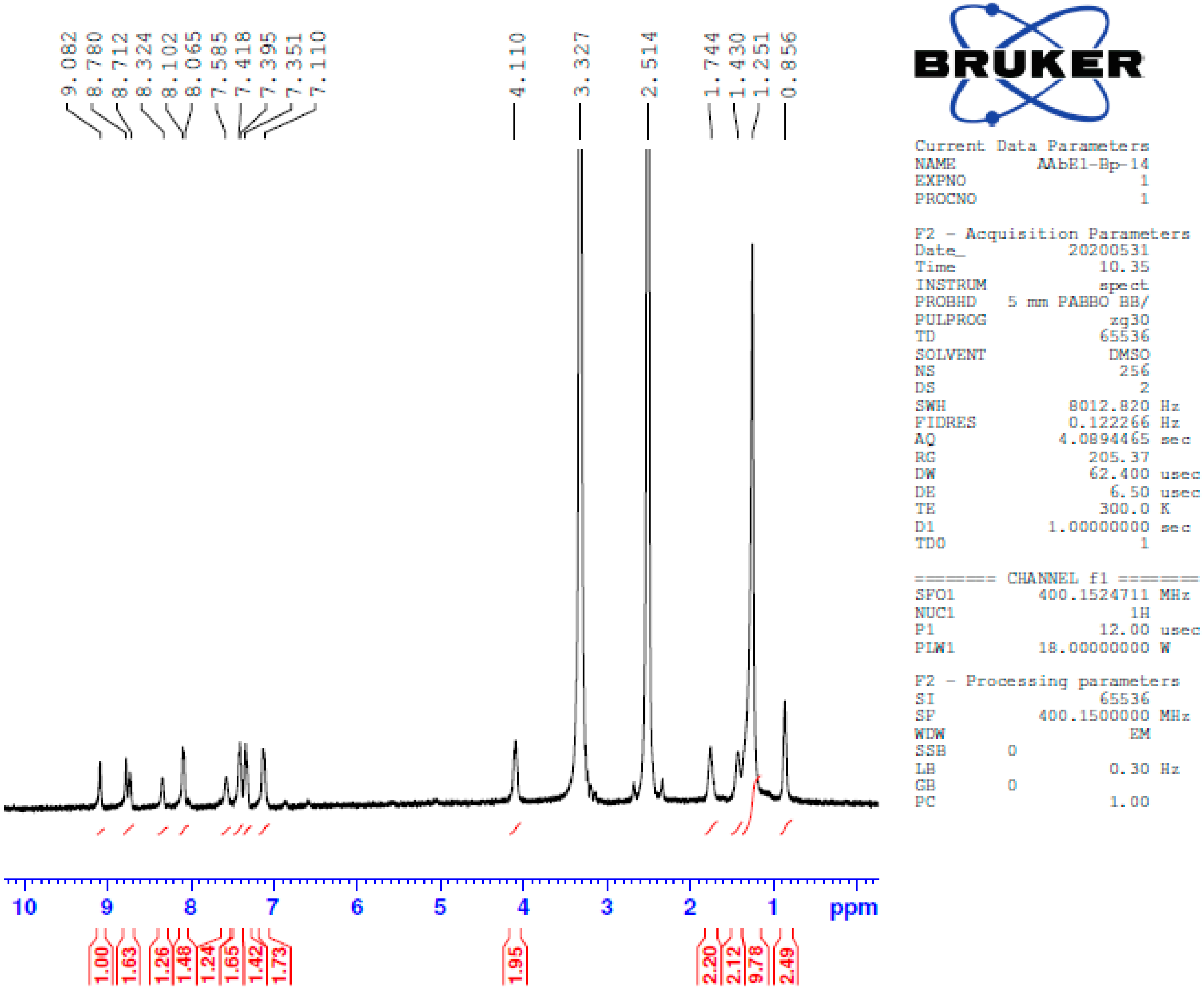

Figure S5.^{1}H-NMR spectrum of T14

V_x = 4.2067 Debye, V_y = -0.2501 Debye, Vz = -0.1095 Debye

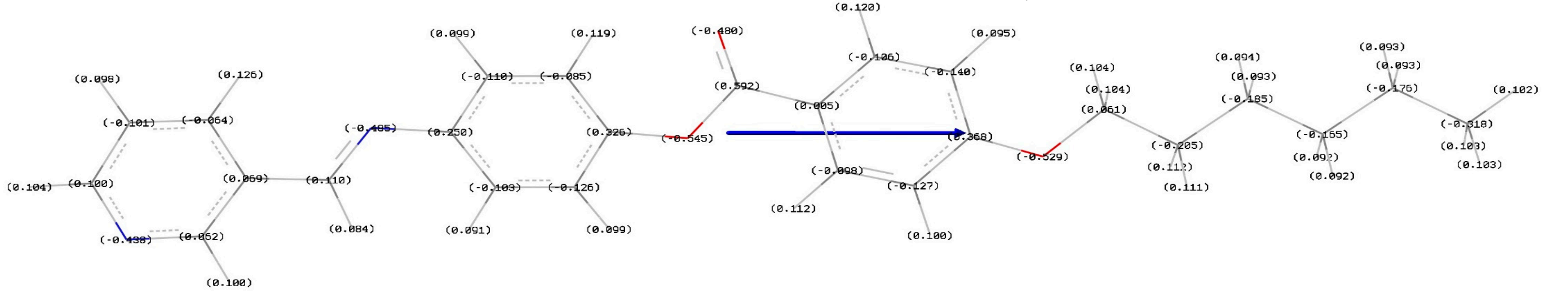

T6

V_x = 4.2539 Debye, V_y = -0.2464 Debye, Vz = -0.0981 Debye

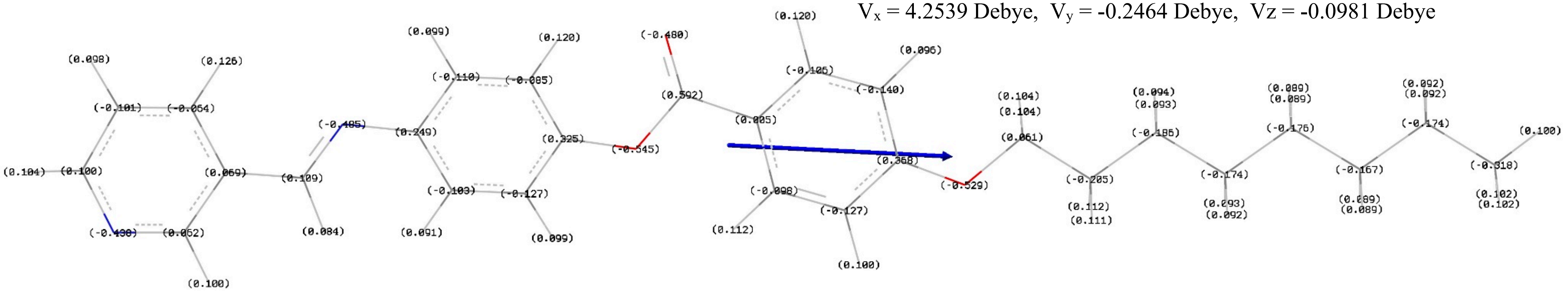

T8

V_x = 4.2792 Debye, V_y = -0.2466 Debye, Vz = -0.0929 Debye

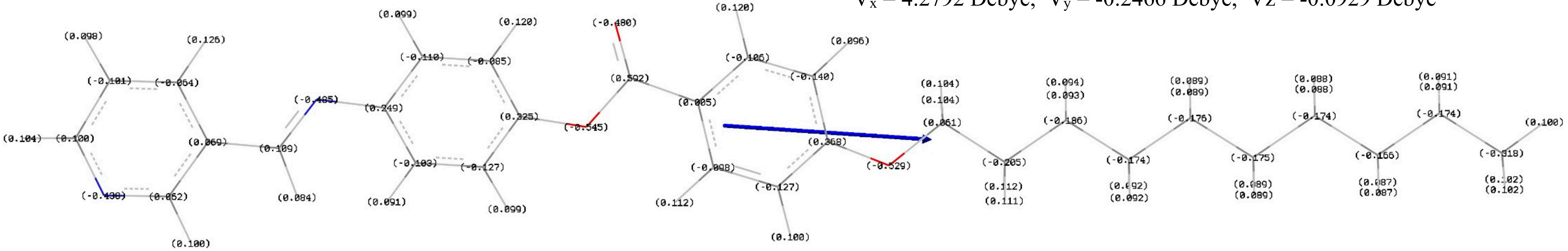

T10

V_x = 4.2938 Debye, V_y = -0.2509 Debye, Vz = -0.0887 Debye

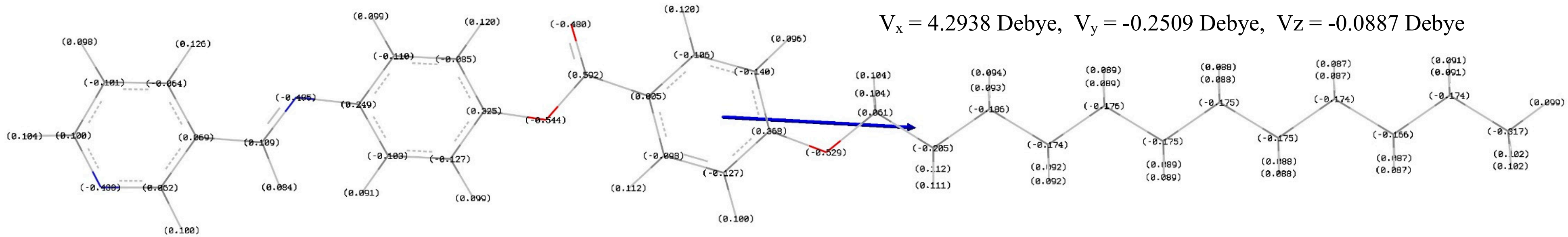

T12

V_x = 4.3025 Debye, V_y = -0.2573 Debye, Vz = -0.0860 Debye

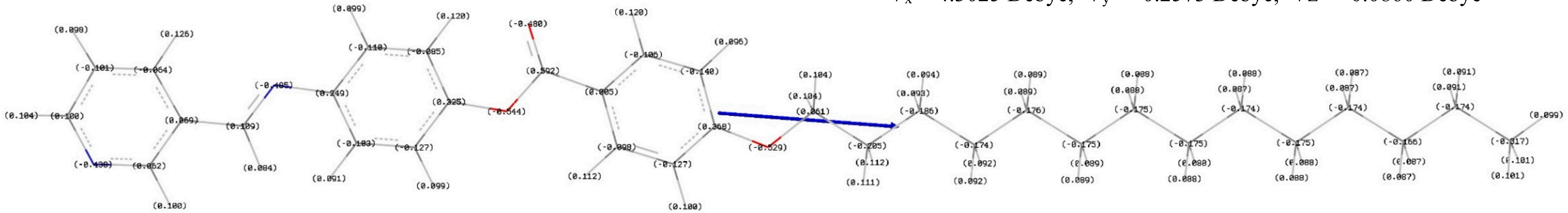

T14

V_x = 4.3081 Debye, V_y = -0.2644 Debye, Vz = -0.0849 Debye

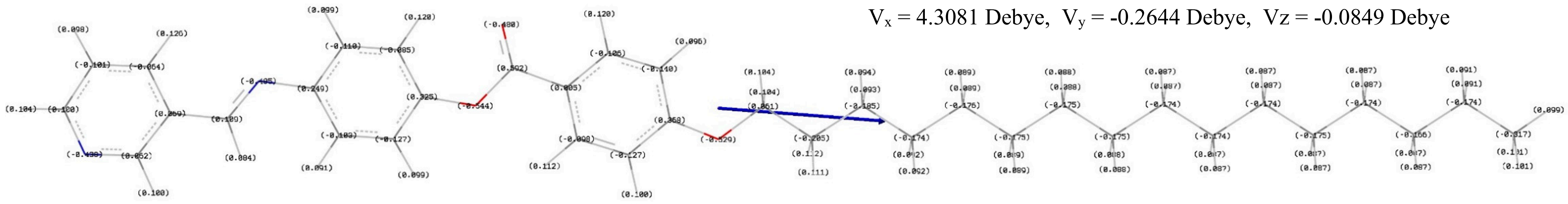

T16

Figure S6. Atomic charges and dipole moment vectors, calculated at B3LYP/6-31G(d,p) level for the **Tn** series
